# The Consolidated Approach to Intervention Adaptation (CLARION): Developing and undertaking an empirically and theoretically driven intervention adaptation

**DOI:** 10.1186/s43058-025-00731-y

**Published:** 2025-05-15

**Authors:** Lydia Ould Brahim, Sylvie D. Lambert, Nancy Feeley, Jane McCusker, Dan Bilsker, Mark J. Yaffe, Rosetta Antonacci, Stephanie Robins, John William Kayser, Christine Genest, Haida Paraskevopoulos, Jessica Blair, Andrea Laizner

**Affiliations:** 1 Ingram School of Nursing, McGill University, 680 Rue Sherbrooke O #1800, Montréal, QC H3A 2M7 Canada; 2 St. Mary’s Research Centre, Montreal, Canada; 3 Centre for Nursing Research & Lady Davis Research Institute, Jewish General Hospital, Montréal, Canada; 4 Epidemiology, Biostatistics and Occupational Health, McGill University, Montréal, Canada; 5 Department of Psychiatry, Faculty of Medicine, University of British Columbia, Vancouver, Canada; 6 Departments of Family Medicine, McGill University, Montréal, Canada; 7 Department of Family Medicine at St. Mary’s Hospital, CIUSSS du Centre-Ouest-de-L’Île-de-Montréal, Montréal, Canada; 8 Department of Nursing, Université du Québec en Outaouais, St-Jérôme, Canada; 9 CIUSSS de l’Ouest-de-l’Île-de-Montréal, Montréal, Canada; 10 Faculty of Nursing Sciences, Université de Montréal, Montréal, QC Canada; 11 St-Mary’s Hospital of the CIUSSS du Centre-Ouest-de-L’Île-de-Montréal, Montreal, Quebec Canada; 12 6915 rue St-Denis, Montreal, H2S 2S3 Canada; 13 Research Institute McGill University Health Centre, Montréal, Canada

**Keywords:** Intervention research, Intervention adaptation, Research design, Depression, Chronic disease

## Abstract

**Background:**

Intervention adaptation, the deliberate modification of the design or delivery of interventions to a new context, is more resource efficient than de novo development. However, adaptation must be approached methodically, as some modifications, such as those to the core components, may compromise the intervention’s initial efficacy. While adaptation frameworks have been published, none have been identified as more likely to result in successful adaptations. Further, frameworks lack the step-by-step details needed for operationalization. Therefore, the goal of this paper is to share our experience in addressing these methodological limitations in intervention adaptation. The objectives were to describe: 1) our development of a step-by-step, theoretically and empirically driven approach to intervention adaptation labelled the ConsoLidated AppRoach to Intervention adaptatiON (CLARION), 2) the application of CLARION in adapting a depression self-management intervention, 3) the facilitators and challenges encountered when using CLARION.

**Methods:**

The development of CLARION was informed by the Medical Research Council guidance, the Method for Program Adaptation through Community Engagement (M-PACE), and a published scoping review identifying the key steps in existing adaptation frameworks. M-PACE was selected for its patient-oriented research principles, its application to a similar complex intervention, and for offering some of the specificity needed for execution. However, the scoping review indicated that M-PACE lacked three critical steps: selecting a candidate intervention, understanding its core components, and pre-testing the adapted intervention. These were added to form CLARION, which was structured in two stages: the first involves selecting an intervention, identifying core components, and deciding on modifications; the second stage solicits interest stakeholder feedback to assess the acceptability of the preliminary adapted intervention (pre-test).

**Results:**

Once CLARION was developed, it was put into action to adapt a depression self-management intervention. CLARION demonstrated several strengths: 1) clearly articulating core components before deciding on modifications, 2) mobilizing a diverse steering committee of experts, including patient partners and developers of the original intervention, which balanced input and efficiency, and 3) establishing committee decision-making rules prior to adjudication (specific criteria and 75% supermajority). Key challenges included defining the types of modifications requiring committee input, determining the extent of the committee’s involvement, and prioritizing the presence of all committee members at meetings to avoid difficulties integrating incongruent feedback.

**Conclusions:**

The development of CLARION contributes to best practices for intervention adaptation by identifying step-by-step guidance as well as facilitators and barriers to its application.

**Supplementary Information:**

The online version contains supplementary material available at 10.1186/s43058-025-00731-y.

Contributions to the Literature • Adapting existing interventions to new contexts may accelerate the translation of research into real-world settings, be less costly than developing new interventions, and reduce research waste by initially assessing fit on a smaller scale.

• Using a framework or systematic process enhances rigour and may be more likely to result in a successful adaptation.

• Although many frameworks exist, they often lack the detail needed to operationalize their steps.

• Using a case example, this paper helps address this gap by describing an adaptation approach we developed called CLARION, detailing how we applied it, and examining what worked well, the challenges encountered, and how to address these challenges.

## Background

Using evidence-informed interventions, defined as those having prior evidence supporting their use, in new contexts (e.g., different geographical settings or populations) is often more resource efficient than developing new ones [1, 2]. This is critical given overburdened healthcare systems facing excessive costs and suboptimal outcomes, as well as research funding becoming increasingly competitive and scarce [3]. Adaptation is defined as the deliberate modification of interventions to improve their fit and enhance acceptability in a new context [4]. To ensure an intervention is feasible and efficacious when applied to a new context, adaptations may be made to content, dosage (intensity), intervention providers (e.g., peers, healthcare professionals), format (e.g., online), and/or procedure (e.g., location, timing) [4, 5].

There is, however, tension between adaptation and fidelity, fidelity being the degree to which an intervention is delivered as designed and maintains its ‘core components’ responsible for its efficacy [4, 6–8]. Adaptation may compromise intervention fidelity by unintentionally diluting the original efficacy, or worse, lead to unanticipated adverse outcomes [1, 6]. This can happen because the ‘core components’ (or active ingredients) of the original intervention, those linked to its mechanisms of action and consequently responsible for its efficacy, might have been changed [1, 4]. One way to address this tension is to use an intervention adaptation framework or approach, which outlines systematic processes that enable interventions to be adapted to fit a new context while minimizing modifications that decrease efficacy [9].

Whereas there is growing understanding of the necessity for intervention adaptation, there is a lack of detail and transparency about processes undertaken (remains a ‘black box’) [8, 10]. To address this, decision-making guides [11] and frameworks have been developed to enhance rigour in adaptation processes [1, 9]. Notably, the Medical Research Council and National Institute for Health and Care Research (MRC-NIHR) Methodology Research Programme published the ADAPT study, which provides overarching consensus-informed guidance on the adaptation of interventions for implementation and/or re-evaluation in new contexts [1]. ADAPT broadly outlines steps and general advice, but it does not concretely detail how to operationalize the proposed steps [4]. Further research on the challenges faced while planning and executing an intervention adaptation will provide critical information for future work [10].

Given this, our objectives were to: 1) detail our development of a theoretically and empirically driven adaptation process, called the ConsoLidated AppRoach to Intervention adaptatiON (CLARION), 2) describe its use in adapting a previously tested self-management depression intervention for adults with chronic conditions, to include a caregiver role, and 3) outline the facilitators and challenges encountered in applying CLARION. In line with this, the methods section will focus on the context and rationale for developing CLARION. Subsequently, in the results section, we delineate the key steps involved in CLARION and use our team’s adaptation to illustrate its application. To support future use of CLARION or similar approaches to adaptation, we include discussion of the facilitators and challenges encountered by our team throughout the process. Finally, in the discussion, we contextualize these considerations within the broader literature on intervention adaptation and suggest ways in which the challenges could be mitigated.

## Methods

### Context: Intervention to adapt

Our team set out to adapt an existing depression intervention for primary care patients with chronic physical health conditions and concomitant depressive symptoms, called the Depression Intervention via Referral, Education, and Collaborative Treatment self-care (*DIRECT-sc*) toolkit [12, 13]. The aim was to include a prescribed role for their caregivers (unpaid family members or friends). Focused on a self-management approach to depression care, the *DIRECT-sc toolkit* provides informational support and strategies based on principles of cognitive behavioural therapy designed to increase self-efficacy in managing depression. The toolkit includes: 1) an anti-depressant skills workbook and audiobook with depression management skill building exercises (e.g., problem-solving) [14], 2) mood monitoring worksheets, and 3) a DVD of testimonials [12, 15]. In previous studies, the toolkit was designed to be used in a self-directed format or in conjunction with telephone guidance. Feedback from caregivers of participants in a previous trial indicated a desire to better understand how they might best support the care recipient in using *DIRECT-sc* [16, 17]. Based on this, we aimed to develop a dyadic version of the *DIRECT-sc* toolkit for care recipients and their caregivers, called *DIRECT-support*.

The decision to undertake this adaptation was also based on literature indicating the considerable deleterious impact of depression on both those with chronic physical diseases as well as their caregivers [18–20]. Further, care recipients and caregivers navigate the emotional impact of chronic disease as an interdependent system [21, 22] suggesting that intervening with both may have more positive impact than focusing on either person alone. Including caregivers in interventions for adults with chronic diseases has also been shown to positively impact adherence and reduce attrition, thereby potentially optimizing the interventions’ beneficial effects [16, 23].

### Approach to developing CLARION: Selecting existing adaptation frameworks

The available MRC guidance on the development and evaluation of complex interventions informed the overall approach to adapting and assessing *DIRECT-support* [24]. Designed to address practical and methodological challenges in developing and evaluating complex health interventions, the MRC framework includes four phases: 1) development, 2) feasibility and piloting, 3) evaluation, and 4) implementation [24]. While intervention adaptation may be more efficient than development, the guidance does not outline specific steps in adaptation (to be undertaken prior to pilot testing). To address this, we reviewed the frameworks for adapating complex health interventions identified in a two previously published reviews; one systematic [4] and a scoping review of frameworks for adapting public health interventions [9]. We selected the Method for Program Adaptation through Community Engagement (M-PACE) [10] to guide our adaptation because it was centered on principles of patient-oriented research, was developed to adapt a complex self-management intervention, and was one of the only approaches that provided a step-by-step description of the adaptation process.

The scoping review by Escoffery et al. [9] identified 8 steps commonly used across existing frameworks (hereafter referred to as ‘8 common steps’), some of which were not included in M-PACE [9, 25]. Most notably, the 8 common steps emphasized the process of selecting the original intervention to adapt, understanding the underlying theory and core components, and included a pre-test of the preliminary adapted intervention (soliciting interest holder feedback) [9, 10]. Incorporating a focus on identifying a candidate intervention and its core components aligns with the ADAPT guidance [1]. Additionally, soliciting feedback on a preliminary version is consistent with MRC guidance, which emphasizes an iterative process of refining interventions based on user input during the development or adaptation phases [24]. Thus, these were included in CLARION. Table 1 summarizes the steps and contribution of the 8 common steps and M-PACE to the adaptation approached we developed labelled CLARION [10, 26]. Given our purpose, steps related to conducting a pilot test, implementation, and evaluation (part of step 6, as well as 7 and 8) were not included in CLARION.

**Table 1:** The Consolidated Approach to Intervention Adaptation (CLARION)

8 Common Steps	5 Steps in M-PACE	Overlap with 6 CLARION Steps
**Assess community or population of interest** - Assess community context, risk factors, and organizational capacity for implementation		8CS:** Assemble a steering committee of experts** (include members with knowledge of community and organizational factors relevant to intervention adaptation and implementation)
**Understand existing evidence-based interventions** - Identify and review relevant evidence-based interventions and understand the theory/logic behind them and their core components		8CS:** Select a candidate intervention** 8CS:** Identify its mechanisms of action and core components**
**Select an intervention matching the new population and context**		
**Decide what components to adapt** - Test the intervention with the new population and interest holders to identify needed adaptations and determine differences between original and new target population - Preserving fidelity to core components and aligning with the new context	**Assemble an adaptation steering committee** - Include researchers, implementers/practitioners, and community members, including at least one expert on the intervention's theory and effectiveness **Implement the unadapted intervention** - Provide participants (from the target population for the adapted intervention) with the unadapted intervention **Solicit feedback from target** participants on optimal adaptation of intervention components - Recommend triangulation of multiple techniques (e.g., surveys, interviews) **Summarize target participant feedback** - Distribute summary to steering committee members	MPACE:** Assemble a steering committee of experts** 8CS + MPACE:** Implement the original (unadapted) intervention with target users and HCPs working with this population** 8CS + MPACE:** Identify and consolidate potential modifications**
**Adapt the original intervention** - Collaboratively adapt content through pilot testing while maintaining core components	**Adjudicate recommendations** - Use three criteria: importance, feasibility, and congruence with unadapted intervention - Prioritizing experiential knowledge of target users, committee seeks consensus on whether to adapt the intervention based on recommended modifications - Subcommittee implements agreed upon modifications	MPACE:** Steering committee review of potential modifications based on adjudication criteria and integrating agreed upon modifications**
**Test the adapted intervention** - Pre-test adapted materials with interest holders and conduct readability tests - (beyond current scope) Conduct pilot test of the adapted intervention in new target population and modify further, if needed		8CS:** Assess acceptability (pre-test) adapted intervention with interest holders (Phase 2)**
Implement the intervention - Develop an implementation plan including identifying implementers, behaviors, outcomes, scope, and instructions - Execute adapted intervention	*Beyond current scope; to be completed after adaptation*	
Evaluate - Document and evaluate the adaptation process - Evaluate the outcomes of the implemented adaptation - Plan evaluation (questions, data collection, analysis, reporting)	*Beyond current scope; to be completed after adaptation*	

8 Common steps were those identified in at least five of the 13 identified frameworks. Shaded rows = Final steps in 8 common ones [part of (6) conducting a pilot test, (7) implement the intervention, and (8) evaluate the adaptation process and outcomes] are excluded because they take place after adapting the intervention and are thus beyond the scope of the current study. Adapted from: 1. Escoffery, C., et al. (2019). A scoping study of frameworks for adapting public health evidence-based interventions. *Translational Behavioral Medicine, 9*(1), 1–10. 2. Chen, E. et al. (2013). Tailoring evidence-based interventions for new populations: a method for program adaptation through community engagement. Eval Health Prof, 36(1), 73–92

8CS = Contributed fromstep identified in 8 common steps [9]; MPACE = Contributed from step identified in Method for Program Adaptation through Community Engagement [10]

## Results

**Fig. 1:**
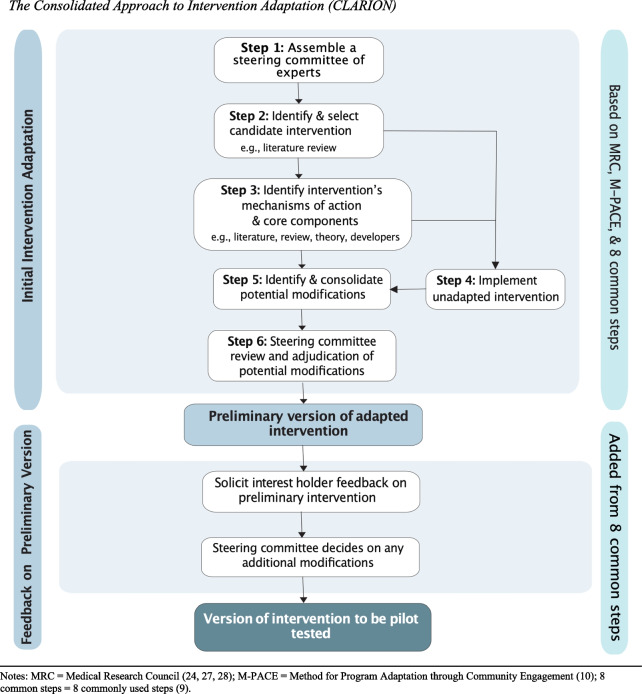
The Consolidated Approach to Intervention Adaptation (CLARION). Notes: MRC = Medical Research Council [24, 27, 28]; M-PACE = Method for Program Adaptation through Community Engagement [10]; 8 common steps = 8 commonly used steps [9]

In combining MRC guidance, M-PACE, and the 8 common steps, CLARION was designed to include two phases: initial adaptation and then an assessment of acceptability of the preliminary adapted intervention (Fig. 1). Given the objectives of this paper, we focus on all steps in Phase 1 including how our team operationalized these as well as the facilitators and challenges encountered while applying CLARION. The six steps in Phase 1 are: 1) assembling a steering committee of experts, 2) selecting a candidate intervention, 3) identifying its mechanisms of action and core components, 4) implementing the original (unadapted) with target users and HCPs working with this population, 5) identifying and consolidating potential modifications, and 6) steering committee review of potential modifications based on adjudication criteria (see Fig. 1).

### Step 1: Assembling a steering committee of experts

In undertaking this step, we assembled a steering committee consisting of 11 experts: care recipient and caregiver partners (also referred to as ‘patient partners’), HCPs working with the target population in settings in which the intervention could be implemented, and researchers, including those involved in the development and evaluation of the *DIRECT-sc toolkit* [13, 14]. Notably, many members had experience across these groups (e.g., as caregiver-researchers or HCPs-researchers). HCPs and researchers were not compensated for their time. Care recipient and caregiver partners were compensated following published guidelines [29].

### Step 2: Identifying and selecting the candidate intervention

To inform this step, a review of online depression self-management interventions [30] and two meta-analyses were conducted: one focused on individual depression self-management interventions for adults with chronic diseases, and the other on non-pharmacological depression interventions for caregivers of those with chronic diseases and co-occurring depressive symptoms [31, 32]. These contributed to 1) confirming whether an existing intervention met the need for a dyadic depression self-management intervention for the target population, 2) examining the effects of similar existing interventions to confirm the merit of proceeding with the adaptation, 3) identifying core components or potential moderators of intervention effect, and 4) assessing the quality and suitability of the selected candidate intervention [30]. The findings of both meta-analyses established that no existing intervention met the identified gap and supported the choice of the *DIRECT-sc toolkit* for adaptation. However, conducting these reviews was time and resource intensive, spanning over 18 months and required team members to have expertise in conducting systematic reviews.

### Step 3: Identifying the intervention's mechanisms of action and core components

The results of the meta-analyses indicated that the self-management skills of decision-making, taking action, and, to a certain extent, problem-solving moderated intervention effects [31, 32]. As such these were identified as core components of *DIRECT-support*. The core components were further described by two theories selected by the team: the Individual and Family Self-Management Theory (IFSMT) [33] and 2) Social Cognitive Theory [34, 35]. IFSMT provides a framework for understanding mechanisms of action of dyadic or family self-management interventions. Social Cognitive Theory outlines mechanisms of action through which individuals engage in and learn health behaviours [35–37]. Both theories include a focus on self-efficacy as well as social support in behaviour change. In consultation with the developers of the original intervention, the mechanisms of action specified in these theories were linked to content in the original intervention (see supplemental material 1 for further detail). Thus, these content areas were retained and not modified (or modified as minimally as possible) in the adaptation.

This step was made easier as the original developers with knowledge of the mechanisms of action were committee members. A clear logic model is critical when deciding on modifications. As there was concern about removing content responsible for the intervention's efficacy, nearly all modifications carried forward were additions, which led to increased intervention content.

### Steps 4 and 5: Implementing the unadapted intervention with interest holders and identifying and consolidating potential modifications

For this step, a qualitative descriptive study with interest holders (dyads and healthcare professionals) was conducted to identify suggested modifications to the *DIRECT-sc toolkit* to include caregivers. Dyads of adults with chronic physical diseases and concomitant depressive symptoms and their caregivers (*n* = 4; 8 individuals) were provided the *DIRECT-sc toolkit* to use. Healthcare professionals (HCPs) working with this population (*n* = 13) also reviewed the toolkit. Participants completed semi-structured interviews to provide feedback on the modifications needed to adapt the intervention to include caregivers. Content analysis [38] of transcripts identified 35 unique potential modifications (findings will be published separately).

### Step 6: Steering committee adjudication of potential modifications

Due to Covid-19 restrictions and varied geographic locations, all three meetings, held over a period of three months, were virtual. The meetings were moderated by the 1st author, who did not vote on the suggested modifications. Given the relatively large number of people on the steering committee, it was difficult to convene all members at the same time. For those who were unable to attend a committee meeting, individual ones with the facilitator were scheduled. However, this resulted in redundancy, loss of discussion among committee members, as well as challenges integrating divergent feedback when raised outside the of the group meetings. When possible, disagreements arising in individual meetings were addressed at the next committee meeting. We tried to address this by scheduling meetings well in advance.

The first meeting served as an introduction; members then discussed and agreed upon the decision-making rules for adjudication with the remaining time spent on adjudication (detail below). The committee agreed that each suggested modification would be adjudicated along three criteria defined in M-PACE [10]: 1) *importance* for effectiveness and reach of the intervention, 2) *feasibility* from interest holders’ perspectives, and 3) *congruence*, meaning how proposed modifications may enhance or interfere with the intervention’s core components. In the case of disagreement, members also approved that congruence with the core components of *DIRECT-sc* would be prioritized. To help guide decisions related to congruence, a summary of the consolidated findings from the meta-analyses as well as theory including a description of the core components of *DIRECT-support* and examples of potential misalignments was circulated prior to the first meeting (see supplemental materials 2). An example of when this arose was in adjudicating the suggested modification of adding an online group chat feature to the toolkit so that people could speak with peers. In addition to feasibility challenges (e.g., difficult to monitor), incongruence with the mechanisms of action of the original intervention was flagged as a concern; committee members cited previous experiences where group chat features had led to negative encounters for participants (incongruent with increasing self-efficacy) as well as sharing information or strategies that may be harmful.

A summary of potential benefits and drawbacks of different decision-making rules (e.g., consensus, simple majority) was provided [1]. How dissenting opinions might be integrated, how ‘voting’ would take place, whether a veto option was needed were explicitly addressed. While M-PACE indicates that steering committee decisions should adhere to the rule of consensus [10], it was decided that disagreements would be discussed, and that, to potentially expedite the adjudication process, a supermajority of 75% agreement across members would be acceptable.

To help streamline discussion, an online survey outlining the 35 proposed modifications derived from Step 4 was sent to committee members prior to the first and second meetings. They were asked to provide feedback on each of the proposed modifications based on the three agreed upon criteria (see supplemental material 3 for example survey) [10]. Committee members were also offered the opportunity to share further feedback via email, and individual meetings were scheduled with those unable to attend group ones. One caregiver partner chose to meet with the facilitator individually rather than participate in group meetings.

Prior to the first meeting, 10 of the 35 suggested modifications reached 100% consensus on the online survey and 16 met the 75% threshold. These 16 items were discussed to allow space for disagreement to be elaborated, all were minor and resolved through discussion. The remaining 9 items were recirculated to the team before the second meeting. The results were again aggregated and presented. At the second meeting, 5 more items reached 75% agreement and were briefly discussed. Adjudication of the remaining 4 items could not be completed in the time allotted for the second meeting, so these were carried forward to the third and final meeting, during which agreement was reached on all outstanding suggested modifications.

Overall, it was agreed that 27 modifications would be carried forward and 8 were not, primarily for reasons of feasibility. Nearly all the retained modifications were additional modules or refining existing content and fidelity consistent (further detail will be published separately) [8]. Over an 11-month period, these modifications were integrated into the *DIRECT-sc toolkit,* and a preliminary version of *DIRECT-support* was then assessed for acceptability.

We found that the composition of the committee and the number of members (*n* = 11) was appropriate for balancing diverse perspectives with efficiency of the adjudication process [10]. Three committee members had extensively worked on *DIRECT-sc* and were able to provide valuable insights on congruence. Most committee members had previously worked with one another, which facilitated communication of differing opinions.

The use of a survey prior to meetings was effective in streamlining discussion by identifying modifications that were already agreed upon by committee members. This helped setting meeting priorities and anticipating how to allocate time. However, committee members felt that adjudicating 35 modifications along three criteria was overly complicated. Further, the meaning of the three criteria (importance, feasibility, and congruence) were not clear to all committee members. Based on this, the survey was updated so that respondents could state simply whether they are agreed or disagreed that the modification would 1) be helpful/useful and 2) be possible/feasible. Only one member of the committee completed this updated version. This was because the caregiver partner joined the committee after the other members had already completed the original survey and shared this feedback. This person reported that the new version was clear and straightforward to complete. Examples of both versions of surveys are included in supplemental material 3. In our process, we shared anonymized online survey results at the beginning of each meeting. Reflecting on this experience, committee members reported that it could have be beneficial to circulate this information prior to meetings.

## Discussion

To address the need for concrete guidance when undertaking an intervention adaptation, we proposed the ConsoLidated AppRoach to Intervention adaptatiON (CLARION) and described its key steps along with how these were operationalized in the context of adapting *DIRECT-support*, a self-management depression intervention. Drawing from our team's experience, key issues included the following: 1) clarifying the mechanisms of action and core components of an intervention, 2) timing of committee feedback, 3) meeting facilitation, 4) streamlining CLARION, 5) including the developers of the original intervention, and 6) integrating care recipient partners into the steering committee of experts.

### Identifying ‘Core Components’

A critical gap in the current adaptation literature is a lack of theorizing about the role of interventions’ mechanisms of action. Until more recently, how an intervention’s ‘core components’ are linked with its mechanisms of action is rarely made explicit [4, 11]. When addressed, core components are typically determined based on underlying theory (e.g., of behaviour change), which outlines stipulated theoretical relationships among intervention components/activities, mediators of change, and outcomes [4, 39, 40]. Complex interventions’ core components are not always empirically established, given the many obstacles to this [5, 39, 41]. It may be possible to begin addressing this gap by integrating intervention logic models, findings of systematic reviews and meta-analyses, and clearly outlining theoretical foundations of interventions’ mechanisms [40]. This may also be achieved by examining the theorized mechanisms of action in trials [41].

In addition, The Human Behaviour-Change Project (HBCP) is seeking to address this challenge using machine learning to extract, synthesize, and interpret findings of behaviour change intervention evaluations [42]. The aim is to improve predictions of intervention outcomes in novel scenarios [43]. To better understand why interventions have (not) worked, a branch of the project is focused on developing a classification system to describe intervention mechanisms of action [44]. This common vocabulary will be useful in aggregating and comparing data across sources and underpins the larger goal of the project to predict effect sizes for combinations of intervention, populations, settings, and target behaviours [44, 45]. Access to such a system may greatly advance the success of behavioural intervention adaptation to new contexts.

### Timing of Committee Feedback

Feedback from the steering committee was that their input should occur at three points: 1) when initially presented with the suggested modifications to adjudicate, 2) when reviewing how agreed upon modifications were integrated into the preliminary version of the adapted intervention, and 3) after acceptability data on the preliminary version was collected (not included in Phase 1). It was also suggested that the specifics regarding how many iterations of feedback (e.g., how many versions of the preliminary adapted intervention would be shown to the committee prior to moving forward) and the procedure (email, group or individual meetings) should be discussed beforehand. In our case, all initial adjudication on the suggested modifications took place at meetings; however, the preliminary version of the adapted intervention was circulated by email and feedback was sought through this medium. This was not specified in advance and though no major issues arose, we suggest that the frequency and mode of communication (e.g., meetings, email) for feedback by committee members be clearly delineated ahead of time to pre-empt potential misunderstandings and to provide a clear sense of what committee membership will entail.

### Steering Committee Meeting Facilitation

We found it important to prepare meeting facilitation strategies ahead of time including how to address the challenge of balancing time efficiency with space for dialogue. In future work, it may also be worth considering strategies for how to respond or move forward if there is staunch disagreement. As there may be varying comfort levels among committee members speaking during meetings, we propose using several strategies to create space for members to share opinions. These can potentially include an anonymous survey, offering the option of direct messaging the facilitator (if meeting online), or group communication strategies like ‘go arounds’ in which each person is given time to speak on a specific issue or highlight an item that was notable to them [46, 47]. Based on our experience, we also suggest that any individual feedback be addressed (perhaps anonymously) at committee meetings to allow for deliberation among all members.

### Streamlining CLARION

In our case, the meta-analyses were the most time and resources consuming part of CLARION. It is likely necessary to review the literature to confirm the need for an adaptation as well as identify a candidate intervention. However, meta-analyses might not be required if the mechanisms of action and core components of the intervention to be adapted are known. Prioritizing this when selecting a candidate intervention is likely to decrease the time and resources required for adaptation.

We also chose to conduct individual or dyadic interviews with interest holders, however, focus groups may be more resource-efficient while still providing needed information [48, 49]. Though attention must be paid to eliciting input from those who may be less comfortable speaking in group settings, focus groups may have the advantage of synergy between participants increasing the generation of new views or helping participants clarify their views [48, 49].

As noted, our steering committee convened three times, supplemented by additional individual meetings as needed. All suggested intervention modifications were aggregated and presented to the committee for discussion. Members reported that agreeing on minor modifications (e.g., editorial, aesthetic) unnecessarily prolonged meetings. Several strategies could be used to potentially streamline committee decisions. The scope of modifications requiring committee approval could be narrowed (e.g., excluding minor revisions). Having all committee members attend a single longer, synchronous meeting rather than having multiple meetings as well as individual ones could avoid potential redundancy and be more time-efficient overall; In the case of staunch disagreement, one way forward could be by collecting acceptability and feasibility data on the issue during the next phases of the project to inform decision-making. Agreeing on the types of modifications that require committee consideration and how much input members would have at different decision-making points throughout the process could improve efficiency. In addition, a decision tree called Decision-making for Evaluation of Adaptations (IDEA) has been developed; it offers options at critical decision-points that may be useful in tailoring the adaption process to one’s specific context and needs [11].

### Including the Original Developers

The benefits of including the developers of the original intervention remains a point of debate within the intervention adaptation literature [2]. Reported benefits include having additional information to understand the mechanisms of action of the intervention in the new context that may not have been described in publications. Conversely, the literature identifies potential challenges including conflicts of interest (e.g., financial or pressure to demonstrate positive outcomes), power imbalances, and emotional investment in the work [2].

In our case, developers of *DIRECT-sc* on the steering committee did, as indicated, provide much insight regarding its mechanisms of action. In discussing the suggested modifications these committee members provided valuable information regarding avenues that had previously been explored (e.g., difficulties maintaining up-to-date resource lists, endorsing/vetting these). This information was critical in assessing the feasibility of many of the suggested modifications. No obstacles related to conflict of interest were noted. However, this may be because *DIRECT-sc* has been made publicly and freely available. To prevent or counter potential issues, suggested strategies include early discussion about roles and the possibility that developers may need to recuse themselves from certain decisions or that possible conflicts must be clearly documented. Issues concerning intellectual property rights should be explored as early as possible [2].

### Integrating Care Recipient Partners

A growing body of literature explores the role of care recipient partners in health research and provides helpful insight into operationalizing these partnerships to benefit those involved [50–53]. For example, in initially discussing involvement it is critical to specify the purpose of and motivation for the engagement and expectations, including roles and level of responsibility [54, 55]. Other logistics such as duration of the project and number of anticipated hours as well as compensation should be discussed openly and agreed upon early on [55]. A recent survey of care recipient partners (often referred to as patient partners) found that only half reported feeling adequately compensated in their role [56]. Further studies also indicate that a lack of financial compensation may exacerbate power imbalances between researchers and care recipient partners, a barrier in taking on the role [56]. However, if provided, compensation can positively impact one’s contributions feeling valued [50, 52].

Offering trainings to care recipient partners is another possible avenue for addressing potential power imbalances. Generally, care recipient partners have reported that trainings are appreciated and increased their confidence and ability to contribute [50]. However, trainings to support the meaningful integration of patient partnership into a research project are recommended for all team members, including researchers and HCPs [52, 53, 57].

Another recommendation is adapting the level of engagement to the needs of care recipients’ partners [50, 53]. In our case, one partner was comfortable attending the committee meetings and the other preferred to meet with the facilitator individually and less frequently. However, contrary to recommendations, this resulted in the care recipient partners not interacting with one another and only one attending committee meetings, placing this person in the position of being the sole voice for this group. Relationships among patient partners have been reported to be one of the most helpful supports in undertaking this role along with having a contact person and access to training [56]. This issue may have been less pronounced in the application of CLARION as the care recipient partner who attended meetings had extensive experience working in research and offered input from both these vantage points.

Several reviews have identified barriers, facilitators, and strategies to support engagement in research and positive patient partner experiences [50–53]. Across studies, a frequent, perhaps unsurprising, finding is that feeling one has meaningfully contributed is critical to having a positive experience [50]. Most commonly, patient partners report that their reasons for engaging in this work relate to a desire to help others by improving healthcare and research [50, 56]. Seeing the impacts of one’s contributions on the study and decisions taken is one way to attend to this motivation. A strategy recommended to achieve this is providing specific feedback on the ways in which all team members’ input has been integrated into the project [50]. It may also be important to highlight how the study fits into a broader picture of advancing or improving healthcare.

### Limitations and Future Research

We have endeavored to provide detail on the rationales for developing CLARION as well as how it was operationalized. This has included areas for improvement that may be beneficial to future research, however, there are several overarching limitations worth highlighting. Most notably, we do not have data addressing whether using CLARION results in more successful adaptation as compared to alternative existing approaches. In looking across the literature, the aim was to develop an approach informed by the best available guidance, emphasizing patient-centered principles while addressing potential gaps in existing frameworks. However, research across diverse settings and interventions is needed to determine if CLARION offers advantages or results in more ‘successful’ adaptations. Future work may also indicate whether it may be most useful in particular contexts and/or which steps are critical, and which can potentially be abbreviated.

It is worth noting that sociodemographic data were not collected to describe the characteristics of the steering committee. Nine of the 11 members were invited to participate related to their professional experiences (e.g., HCPs, researchers) and were therefore not representative of the general population. The committee adjudicated based on feedback collected from study participants who were end users of *DIRECT-support *or HCPs. Recruitment of these participants targeted diverse demographics.

## Conclusion

We have described the development of CLARION, an empirically and theoretically driven intervention adaptation approach as well as presented an example of how it was operationalized. To address a gap in the literature, we have detailed actionable steps as well as the facilitators and challenges encountered in using CLARION. We have sought to provide guidance for future research and support the growing work seeking to identify best-practices for intervention adaptation.

## Supplementary Information

Supplementary Material 1.

Supplementary Material 2.

Supplementary Material 3.

## Data Availability

This study and analysis plan were not formally registered. The study is based on findings of two systematic reviews and qualitative studies collecting and analyzing interview data collected from stakeholders (IRB CIUSSS-Ouest Montreal, #16–42). The systematic reviews (referenced within the main text) have been published separately, were registered, and the related data are available via these publications. Transcribed interview data cannot be fully de-identified and with therefore not be made public. If possible, de-identified data from this study will be made available (as allowable according to institutional IRB standards) by emailing the corresponding author. Materials used to conduct the study (e.g., survey and presentation examples) are available as supplemental data and/or by contacting the corresponding author. There is no analytic code associated with this study.

## Abbreviations

MRC-NIHR: Medical Research Council and National Institute for Health and Care Research
CLARION: ConsoLidated AppRoach to Intervention adaptatiON
*DIRECT-sc*: Depression Intervention via Referral, Education, and Collaborative Treatment Self-Care
M-PACE: Method for Program Adaptation through Community Engagement
IFSMT: Individual and Family Self-Management Theory
HBCP: Human Behaviour-Change Project
