# Supplementary material for: The Consolidated Approach to Intervention Adaptation (CLARION): Developing and undertaking an empirically and theoretically driven intervention adaptation
Source: Implement Sci Commun. 2025 May 15;6:59. doi: 10.1186/s43058-025-00731-y (PMC12083050; doi:10.1186/s43058-025-00731-y)
Supplement: Supplementary file 1 — Supplementary Material 1. [file 43058_2025_731_MOESM1_ESM.pdf]

Supplemental Material 1
Identifying EBI Mechanisms of Action and Core Components

Table 3
Identifying Mechanisms of Action and Core Components of the Evidence-based Intervention (EBI)

**Systematic review of self-management interventions for adults with chronic disease(s)** (Ould Brahim et al., 2021)	**Systematic review of non-pharmacological depression interventions for caregivers of those with chronic disease and depressive symptoms** (Lambert et al., 2021)	**Social Cognitive Theory** (Bandura, 1986)	**Individual and Family Self-management Theory** (Ryan & Sawin, 2009)
**Key Skills/Strategies**			
Taking action	Taking action	Goal setting	Goal setting
Decision-making	Decision-making	Reviewing progress	Decision-making
	Problem-solving	Problem-solving	Action planning
		Mastery experience	Self-monitoring
		Vicarious experience	Social support
		Verbal persuasion	Negotiated collaboration
		Physiological Feedback	Disease information

Notes: Overlap among the sources of evidence (theories and systematic reviews) was interpreted as the core components of the intervention. Green highlight indicates core components identified across three sources of evidence and yellow highlight those identified across two. Those highlighted were therefore retained (or modified as minimally as possible) in the adapted intervention. Cells not highlighted could potentially be modified. **Definitions**: Decision-making - often occurs in the context of problem-solving and is based on having enough and accurate information to make decisions related to illness management (Lorig & Holman, 2003); problem-solving: using a structured approach including learning skills such as defining the problem and generating solutions (Bilsker et al., 2012; Lorig & Holman, 2003; van Grieken et al., 2015); taking action: making a plan and carrying it out, learning skills involved in behaviour change (Lorig & Holman, 2003); self-monitoring: monitoring symptoms and evaluating whether current strategies are working effectively and, when necessary, reassessing treatment plans (Partners). 2003; van Grieken et al., 2015; van Grieken et al., 2014); social support: arranging instrumental and emotional support and involving close friends/family in treatment and support (Michie et al., 2008; van Grieken et al., 2015; van Grieken et al., 2014).

Table 4
Potentially Modifiable (discretionary) EBI Components

**Systematic review of self-management interventions for adults with chronic disease(s)** (Ould Brahim et al., 2021)	**Systematic review of non-pharmacological depression interventions for caregivers of those with chronic disease and depressive symptoms** (Lambert et al., 2021)
**Non statistically significant moderators of depression**	
Mode of delivery (e.g., online, face-to-face)	Mode of delivery (e.g., online, face-to-face)
Length of intervention	Length of the intervention
Duration	Duration
	Level of guidance (self-directed, guided)

Note: Based on the two systematic reviews, blue highlight indicates non-statistically significant moderators of intervention effect on depression in both reviews. These components were considered potentially modifiable in the adaptation (with caution due to small sample sizes).
