# Supplementary material for: The Consolidated Approach to Intervention Adaptation (CLARION): Developing and undertaking an empirically and theoretically driven intervention adaptation
Source: Implement Sci Commun. 2025 May 15;6:59. doi: 10.1186/s43058-025-00731-y (PMC12083050; doi:10.1186/s43058-025-00731-y)
Supplement: Supplementary file 2 — Supplementary Material 2. [file 43058_2025_731_MOESM2_ESM.pdf]

Supplemental Material 2
Summary of the consolidated findings from the systematic reviews and theory presented to the steering committee of experts

Existing Evidence

RESEARCH ARTICLE Open Access

The effects of self-management interventions on depressive symptoms in adults with chronic physical disease(s) experiencing depressive symptomatology: a systematic review and meta-analysis

Check for updates

Lydia Ould Brahim[1*], Sylvie D. Lambert[1,2], Nancy Feeley[1,3], Chelsea Coumoundouros[4], Jamie Schaffler[2], Jane McCusker[2,5], Erica E. M. Moodie[6], John Kayser[7], Kendall Kolne[8], Eric Belzile[2] and Christine Genest[9]

Abstract

Background: Chronic diseases are the leading cause of death worldwide. It is estimated that 20% of adults with chronic physical diseases experience concomitant depression, increasing their risk of morbidity and mortality. Low intensity psychosocial interventions, such as self-management, are part of recommended treatment; however, no systematic review has evaluated the effects of depression self-management interventions for this population. The primary objective was to examine the effect of self-management interventions on reducing depressive symptomatology in adults with chronic disease(s) and co-occurring depressive symptoms. Secondary objectives were to evaluate the effect of these interventions on improving other psychosocial and physiological outcomes (e.g., anxiety, glycemic control) and to assess potential differential effect based on key participant and intervention characteristics (e.g., chronic disease, provider).

Methods: Studies comparing depression self-management interventions to a control group were identified through a) systematic searches of databases to June 2018 [MEDLINE (1946 -), EMBASE (1996 -), PsycINFO (1967 -), CINAHL (1984 -)] and b) secondary 'snowball' search strategies. The methodological quality of included studies was critically reviewed. Screening of all titles, abstracts, and full texts for eligibility was assessed independently by two authors. Data were extracted by one author and verified by a second.

Results: Fifteen studies were retained: 12 for meta-analysis and three for descriptive review. In total, these trials included 2064 participants and most commonly evaluated interventions for people with cancer ($n = 7$) or diabetes ($n = 4$). From baseline to < 6-months (T1), the pooled mean effect size was – 0.47 [95% CI –0.73, – 0.21] as compared to control groups for the primary outcome of depression and – 0.53 [95% CI –0.91, – 0.15] at ≥ 6-months (T2). Results were also significant for anxiety (T1) and glycemic control (T2). Self-management skills of decision-making and taking action were significant moderators of depression at T1.

Springer Link

Systematic Review | Published: 22 June 2021

Non-pharmacological Interventions for Caregivers with Depression and Caregivers of Care Recipients with Co-morbid Depression: Systematic Review and Meta-analysis

Sylvie Lambert RN, PhD, Lydia Ould Brahim RN, MSc, Jane McCusker MD, DrPH, Chelsea Coumoundouros MScPH, Li-Anne Audet RN, MSc, Mark Yaffe BSc, MDCM, MClSc, CCFP, FCFP, John Kayser RN, PhD, Mona Magalhaes MA, Eric Belzile MSc & Nerida Turner BAppSc(OT)

Journal of General Internal Medicine (2021) | Cite this article

154 Accesses | **11** Altmetric | Metrics

Abstract

Background

Caregivers experiencing depression or caring for people experiencing depression are at risk of high burden. This systematic review examined the effect of non-pharmacological interventions for caregivers that (a) target improving caregivers' depressive symptoms, (b) help caregivers [illegible]

Moderate effect [ES -0.47 to -0.53] up to and beyond 6-months post-baseline

Moderate effect [ES -0.62] up to 3-months post-baseline & one dyadic intervention

Existing Evidence

Analysis of moderators of depression in both reviews.

Strong evidence	Decision-making, taking action
Moderate evidence	Problem-solving
Minimal evidence/ Lack of data	Mode of delivery (e.g., online vs face-to-face), length of intervention (minutes of participation), duration (over what period), level of guidance (self-directed, guided)

Core Self-management Skills

1. Decision-making
   - Can be a component of problem-solving or related to the day-to-day decisions that need to be made by those with chronic diseases. Requires having enough and accurate information to make decisions (e.g., what ‘red flags’ require medical attention).
2. Taking action
   - Related to mastery in self-efficacy theory. Making a short-term plan and carrying it out (1-2 weeks of a specific behaviour – MAST).
3. Problem-solving
   - Includes problem definition, generation of possible solutions, solution implementation, and evaluation of results.

(Bilsker et al., 2012; Lorig & Holman, 2003; van Grieken et al., 2015)

Theoretical Frameworks

1. Individual and Family Self-management Theory (Ryan & Sawin, 2009)
2. Social Cognitive Theory (Bandura, 1986)
    - Self-efficacy
    - Collective efficacy (Bandura, 1997)

Social Cognitive Theory	Individual and Family Self-management Theory
Mastery experience (SE)	Self-efficacy (SE)
Vicarious experience (SE)	Self-monitoring
Verbal persuasion (SE)	Social support
Physiological Feedback (SE)	
Social support	

Joint Display Table of Key Information: Theoretical Guidance and Systematic Review

Review of Caregiver Interventions	Review of Individual Interventions	Social Cognitive Theory	Individual and Family Self-management Theory
**Key Skills/Strategies**			
Taking action	Taking action	Goal setting	Goal setting
Decision-making	Decision-making	Reviewing progress	Decision-making
Problem-solving		Problem-solving	Action planning
		Mastery experience	Self-monitoring
		Vicarious experience	Social support
		Verbal persuasion	Negotiated collaboration
		Physiological Feedback	Disease information

**Non-significant moderators of depression**	
Mode of delivery (e.g., online, face-to-face)	Mode of delivery (e.g., online, face-to-face)
Length of intervention	Length of the intervention
Duration	Duration
	Level of guidance (self-directed, guided)

Take Home Messages

The findings of the two systematic reviews and theoretical basis for the intervention support these core components:

1) Three non-negotiable core skills: decision-making, taking action, problem-solving
2) Core principles of delivery central to behaviour change (e.g., vicarious experience, self-monitoring)

Adjudication Process

According to M-PACE, evaluate each suggestion based on:

1. **Importance:** Degree to which it is perceived that the change could improve program effectiveness and reach
2. **Feasibility:** Approached from several perspectives (participants, host sites, etc.)
3. **Congruence:** Working with, working against, or not interfering with the core components of the original intervention (and the theoretical basis, reviews)

(Chen et al., 2013)
