# Supplementary material for: The Consolidated Approach to Intervention Adaptation (CLARION): Developing and undertaking an empirically and theoretically driven intervention adaptation
Source: Implement Sci Commun. 2025 May 15;6:59. doi: 10.1186/s43058-025-00731-y (PMC12083050; doi:10.1186/s43058-025-00731-y)
Supplement: Supplementary file 3 — Supplementary Material 3. [file 43058_2025_731_MOESM3_ESM.pdf]

Initial Version of the Survey

Default Question Block

Steering Committee - Survey 1

The aim of this phase of the study is to adapt the *DIRECT-sc* toolkit (including the Antidepressant Skills Workbook) to include a prescribed role for caregivers. The suggestions below were drawn from interviews with HCPs as well as dyads of care recipients with a chronic disease and depressive symptoms and their caregivers who used or reviewed the toolkit.

Based on the method for program adaptation selected (M-PACE), three criteria are being used to identify needed changes to the *DIRECT-sc* toolkit:

1. Impact on effectiveness and/or reach of the intervention;
2. Feasibility (from participant perspective and/or in terms of implementation); and
3. Congruence with the original intervention (enhancing or not negatively impacting the 'active ingredients').

Below, please state the extent to which you agree that the suggested changes align with or positively impact these criteria. Strongly agree or agree indicate that this change is worth consideration.

If any of the suggestions are unclear to you, please feel free to skip them (some of the phrasing was taken directly from interview data).

The survey is made up of 3 sections with an overall total of 35 suggestions. If you do not have time to answer all of the questions, please skip to the end and click 'submit.'

Thank you so much for your input!

Please enter your name below.

Section 1: Caregiver Content

To what extent do agree with the following suggestions for modifications:

1) Add content for caregivers about how they may also be at risk for depression.

	Strongly Agree	Agree	Disagree	Strongly Disagree
Effectiveness and/or reach of the intervention	○	○	○	○
Feasibility (from participant perspective and/or in terms of implementation)	○	○	○	○
Congruence with 'active ingredients' (core components) of original intervention	○	○	○	○

2) Add content for caregivers on self-care (tips and the importance of it).

	Strongly Agree	Agree	Disagree	Strongly Disagree
Effectiveness and/or reach of the intervention	○	○	○	○
Feasibility (from participant perspective and/or in terms of implementation)	○	○	○	○
Congruence with 'active ingredients' (core components) of original intervention	○	○	○	○

3) Add content on supporting a loved one experiencing depression (e.g., daily support, activities that could be shared, supportive communication).

	Strongly Agree	Agree	Disagree	Strongly Disagree
Effectiveness and/or reach of the intervention	○	○	○	○
Feasibility (from participant perspective and/or in terms of implementation)	○	○	○	○
Congruence with 'active ingredients' (core components) of original intervention	○	○	○	○

4) Add content emphasizing that caregivers can seek help/support (e.g., respite) as well.

	Strongly Agree	Agree	Disagree	Strongly Disagree
Effectiveness and/or reach of the intervention	○	○	○	○
Feasibility (from participant perspective and/or in terms of implementation)	○	○	○	○
Congruence with 'active ingredients' (core components) of original intervention	○	○	○	○

5) Add content for caregivers on what responsibilities they want to take on or boundaries they would like to set with regards to caregiving.

	Strongly Agree	Agree	Disagree	Strongly Disagree
Effectiveness and/or reach of the intervention	○	○	○	○
Feasibility (from participant perspective and/or in terms of implementation)	○	○	○	○
Congruence with 'active ingredients' (core components) of original intervention	○	○	○	○

6) Add content on how depression can impact the whole family.

	Strongly Agree	Agree	Disagree	Strongly Disagree
Effectiveness and/or reach of the intervention	○	○	○	○
Feasibility (from participant perspective and/or in terms of implementation)	○	○	○	○
Congruence with 'active ingredients' (core components) of original intervention	○	○	○	○

7) Add more information on depression for caregivers (how the antidepressant skills help, more background) so that they can help guide the care recipient or answer the care recipient's questions.

	Strongly Agree	Agree	Disagree	Strongly Disagree
Effectiveness and/or reach of the intervention	○	○	○	○
Feasibility (from participant perspective and/or in terms of implementation)	○	○	○	○
Congruence with 'active ingredients' (core components) of original intervention	○	○	○	○

8) Add content to the mood monitoring section to guide or encourage both parties communicate about their mood with each other.

	Strongly Agree	Agree	Disagree	Strongly Disagree
Effectiveness and/or reach of the intervention	○	○	○	○
Feasibility (from participant perspective and/or in terms of implementation)	○	○	○	○
Congruence with 'active ingredients' (core components) of original intervention	○	○	○	○

9) Add content to help caregivers problem-solve about communicating with the care recipient about challenging topics (e.g., if they aren't eating).

	Strongly Agree	Agree	Disagree	Strongly Disagree
Effectiveness and/or reach of the intervention	○	○	○	○
Feasibility (from participant perspective and/or in terms of implementation)	○	○	○	○
Congruence with 'active ingredients' (core components) of original intervention	○	○	○	○

10) Add content on common issues faced by caregivers beyond depression (e.g., financial pressure, burden).

	Strongly Agree	Agree	Disagree	Strongly Disagree
Effectiveness and/or reach of the intervention	○	○	○	○
Feasibility (from participant perspective and/or in terms of implementation)	○	○	○	○
Congruence with 'active ingredients' (core components) of original intervention	○	○	○	○

Section 2: Care recipient-related or general content

To what extent do agree with the following suggestions for modifications:

11) Add more general resources based on region (e.g., support groups for depression or for caregivers, AA, mental health resources, CLSCs).

	Strongly Agree	Agree	Disagree	Strongly Disagree
Effectiveness and/or reach of the intervention	○	○	○	○
Feasibility (from participant perspective and/or in terms of implementation)	○	○	○	○
Congruence with 'active ingredients' (core components) of original intervention	○	○	○	○

12) Add more questions to ask your healthcare team (specific diagnosis, what is my treatment plan, how will my family be involved, etc.) and how to advocate for your needs with your team.

	Strongly Agree	Agree	Disagree	Strongly Disagree
Effectiveness and/or reach of the intervention	○	○	○	○
Feasibility (from participant perspective and/or in terms of implementation)	○	○	○	○
Congruence with 'active ingredients' (core components) of original intervention	○	○	○	○

13) Add more content on 'hope' to help address the feeling that nothing will change and include a reminder that it won't always feel this way and things can be done to help.

	Strongly Agree	Agree	Disagree	Strongly Disagree
Effectiveness and/or reach of the intervention	○	○	○	○
Feasibility (from participant perspective and/or in terms of implementation)	○	○	○	○
Congruence with 'active ingredients' (core components) of original intervention	○	○	○	○

14) Add more information about anti-depressant medication (e.g., side-effects, mixing with alcohol, things to watch out for).

	Strongly Agree	Agree	Disagree	Strongly Disagree
Effectiveness and/or reach of the intervention	○	○	○	○
Feasibility (from participant perspective and/or in terms of implementation)	○	○	○	○
Congruence with 'active ingredients' (core components) of original intervention	○	○	○	○

15) Verify the reading level of the material. If needed, edit so that it is accessible to those with a 5th grade reading level.

	Strongly Agree	Agree	Disagree	Strongly Disagree
Effectiveness and/or reach of the intervention	○	○	○	○
Feasibility (from participant perspective and/or in terms of implementation)	○	○	○	○
Congruence with 'active ingredients' (core components) of original intervention	○	○	○	○

16) Include more tailoring of content to each person's needs.

	Strongly Agree	Agree	Disagree	Strongly Disagree
Effectiveness and/or reach of the intervention	○	○	○	○
Feasibility (from participant perspective and/or in terms of implementation)	○	○	○	○
Congruence with 'active ingredients' (core components) of original intervention	○	○	○	○

17) Change scales throughout the materials so they are all consistent (some 0-100, some 0-10).

	Strongly Agree	Agree	Disagree	Strongly Disagree
Effectiveness and/or reach of the intervention	○	○	○	○
Feasibility (from participant perspective and/or in terms of implementation)	○	○	○	○
Congruence with 'active ingredients' (core components) of original intervention	○	○	○	○

18) In terms of 'reactivating your life,' it may be hard for some people to believe that activity can lead to feeling better. Add sentence with further justification (e.g., studies have shown…)

	Strongly Agree	Agree	Disagree	Strongly Disagree
Effectiveness and/or reach of the intervention	○	○	○	○
Feasibility (from participant perspective and/or in terms of implementation)	○	○	○	○
Congruence with 'active ingredients' (core components) of original intervention	○	○	○	○

19) Add more content on 'normalizing' or de-stigmatizing depression.

	Strongly Agree	Agree	Disagree	Strongly Disagree
Effectiveness and/or reach of the intervention	○	○	○	○
Feasibility (from participant perspective and/or in terms of implementation)	○	○	○	○
Congruence with 'active ingredients' (core components) of original intervention	○	○	○	○

20) Add more content for care recipients about focusing on self-care and overcoming guilt about not being able to do certain things.

	Strongly Agree	Agree	Disagree	Strongly Disagree
Effectiveness and/or reach of the intervention	○	○	○	○
Feasibility (from participant perspective and/or in terms of implementation)	○	○	○	○
Congruence with 'active ingredients' (core components) of original intervention	○	○	○	○

21) Reformulate some suggestions that are negatively framed (do not…) to be more positive (try this, do this, consider this).

	Strongly Agree	Agree	Disagree	Strongly Disagree
Effectiveness and/or reach of the intervention	○	○	○	○
Feasibility (from participant perspective and/or in terms of implementation)	○	○	○	○
Congruence with 'active ingredients' (core components) of original intervention	○	○	○	○

22) Add a more detailed step-by-step introduction on how to use the materials in the toolkit.

	Strongly Agree	Agree	Disagree	Strongly Disagree
Effectiveness and/or reach of the intervention	○	○	○	○
Feasibility (from participant perspective and/or in terms of implementation)	○	○	○	○
Congruence with 'active ingredients' (core components) of original intervention	○	○	○	○

23) Add more encouragement to get further help or support.

	Strongly Agree	Agree	Disagree	Strongly Disagree
Effectiveness and/or reach of the intervention	○	○	○	○
Feasibility (from participant perspective and/or in terms of implementation)	○	○	○	○
Congruence with 'active ingredients' (core components) of original intervention	○	○	○	○

Section 3: Format/Mode of delivery

To what extent do agree with the following suggestions for modifications:

24) As few people were able to watch the DVD/video (it included info from HCPs on depression and testimonials), add written testimonials about depression.

	Strongly Agree	Agree	Disagree	Strongly Disagree
Effectiveness and/or reach of the intervention	○	○	○	○
Feasibility (from participant perspective and/or in terms of implementation)	○	○	○	○
Congruence with 'active ingredients' (core components) of original intervention	○	○	○	○

25) Add more visual (graphic/images) representations of the written content.

	Strongly Agree	Agree	Disagree	Strongly Disagree
Effectiveness and/or reach of the intervention	○	○	○	○
Feasibility (from participant perspective and/or in terms of implementation)	○	○	○	○
Congruence with 'active ingredients' (core components) of original intervention	○	○	○	○

26) Add more 'upbeat' colours.

	Strongly Agree	Agree	Disagree	Strongly Disagree
Effectiveness and/or reach of the intervention	○	○	○	○
Feasibility (from participant perspective and/or in terms of implementation)	○	○	○	○
Congruence with 'active ingredients' (core components) of original intervention	○	○	○	○

27) Add further summaries of key points.

	Strongly Agree	Agree	Disagree	Strongly Disagree
Effectiveness and/or reach of the intervention	○	○	○	○
Feasibility (from participant perspective and/or in terms of implementation)	○	○	○	○
Congruence with 'active ingredients' (core components) of original intervention	○	○	○	○

28) Increase type face (font) size and verify that type face (font) is the most accessible to readers.

	Strongly Agree	Agree	Disagree	Strongly Disagree
Effectiveness and/or reach of the intervention	○	○	○	○

	Strongly Agree	Agree	Disagree	Strongly Disagree
Feasibility (from participant perspective and/or in terms of implementation)	○	○	○	○
Congruence with 'active ingredients' (core components) of original intervention	○	○	○	○

29) Add visual component (like a Powerpoint) to go with the audio version (CD) of the workbook.

	Strongly Agree	Agree	Disagree	Strongly Disagree
Effectiveness and/or reach of the intervention	○	○	○	○
Feasibility (from participant perspective and/or in terms of implementation)	○	○	○	○
Congruence with 'active ingredients' (core components) of original intervention	○	○	○	○

30) Use audio format in which the speed can be adjusted (faster or slower pace).

	Strongly Agree	Agree	Disagree	Strongly Disagree
Effectiveness and/or reach of the intervention	○	○	○	○
Feasibility (from participant perspective and/or in terms of implementation)	○	○	○	○
Congruence with 'active ingredients' (core components) of original intervention	○	○	○	○

31) Develop online version that is more interactive.

	Strongly Agree	Agree	Disagree	Strongly Disagree
Effectiveness and/or reach of the intervention	○	○	○	○
Feasibility (from participant perspective and/or in terms of implementation)	○	○	○	○

	Strongly Agree	Agree	Disagree	Strongly Disagree
Congruence with 'active ingredients' (core components) of original intervention	○	○	○	○

32) Consider online blog or chat group component so people can connect/interact with others with shared experience.

	Strongly Agree	Agree	Disagree	Strongly Disagree
Effectiveness and/or reach of the intervention	○	○	○	○
Feasibility (from participant perspective and/or in terms of implementation)	○	○	○	○
Congruence with 'active ingredients' (core components) of original intervention	○	○	○	○

33) Include an additional workbook with more activities based on the skills presented.

	Strongly Agree	Agree	Disagree	Strongly Disagree
Effectiveness and/or reach of the intervention	○	○	○	○
Feasibility (from participant perspective and/or in terms of implementation)	○	○	○	○
Congruence with 'active ingredients' (core components) of original intervention	○	○	○	○

34) Add an online mood monitoring (mood tracker) that can graphically display trends.

	Strongly Agree	Agree	Disagree	Strongly Disagree
Effectiveness and/or reach of the intervention	○	○	○	○
Feasibility (from participant perspective and/or in terms of implementation)	○	○	○	○
Congruence with 'active ingredients' (core components) of original intervention	○	○	○	○

35) Have online mood tracker followed by healthcare team who can follow-up.

	Strongly Agree	Agree	Disagree	Strongly Disagree
Effectiveness and/or reach of the intervention	○	○	○	○
Feasibility (from participant perspective and/or in terms of implementation)	○	○	○	○
Congruence with 'active ingredients' (core components) of original intervention	○	○	○	○

Do you have any other comments or feedback?

Thank you so much for completing this survey!
Please click the arrow below to submit your responses.

Block 1

Powered by Qualtrics

Default Question Block

Second Streamlined Version of the Survey

Survey of Suggested Changes to the Toolkit

The aim of this phase of the project is to adapt the toolkit you reviewed to include a role for caregivers (so that it can be used by two people). Interviews were conducted with adults with chronic physical conditions (e.g., emphysema, cancer) who have experienced depression or low mood and their caregivers (family members or friends who provide support). Interviews were also conducted with healthcare professionals who support those with chronic conditions. Everyone was asked to use or review the toolkit and suggest changes. Some of the changes they suggested were general and some were more specific about what to add or change for caregivers.

We would like to know what you think of these suggestions. There are two things to consider: 1) Do you believe the change would be helpful or useful and 2) do you think it is possible (feasible) to make this change.

Below are the suggested changes. Please respond with how helpful and how feasible it would be to make the changes. If you respond with 'agree' this means that the change is worth considering.

If any of the suggestions are unclear to you, please feel free to skip them.

The survey is made up of 3 sections with an overall total of 35 suggestions. If you do not have time to answer all of the questions, please skip to the end and click 'submit.'

Thank you so much for your input!

Please enter your name below.

____

Section 1: Caregiver Content

Do you agree with the following suggestions for changing the toolkit?

1) Add content for caregivers about how they may also be at risk for depression.

	Agree	Disagree
This would be helpful or useful	○	○
This would be possible/feasible	○	○

2) Add content for caregivers on self-care (tips and the importance of it).

	Agree	Disagree
This would be helpful or useful	○	○
This would be possible/feasible	○	○

3) Add content on supporting a loved one experiencing depression (e.g., daily support, activities that could be shared, supportive communication).

	Agree	Disagree
This would be helpful or useful	○	○
This would be possible/feasible	○	○

4) Add content emphasizing that caregivers can seek help/support (e.g., respite) as well.

	Agree	Disagree
This would be helpful or useful	○	○
This would be possible/feasible	○	○

5) Add content for caregivers on what responsibilities they want to take on or boundaries they would like to set with regards to caregiving.

	Agree	Disagree
This would be helpful or useful	○	○
This would be possible/feasible	○	○

6) Add content on how depression can impact the whole family.

	Agree	Disagree
This would be helpful or useful	○	○
This would be possible/feasible	○	○

8) Add content to the mood monitoring section to encourage both parties communicate about their mood with each other.

	Agree	Disagree
This would be helpful or useful	○	○
This would be possible/feasible	○	○

9) Add content to help caregivers problem-solve about communicating with the care recipient about challenging topics (e.g., if they aren't eating).

	Agree	Disagree
This would be helpful or useful	○	○
This would be possible/feasible	○	○

10) Add content on common issues faced by caregivers beyond depression (e.g., financial pressure, burden).

	Agree	Disagree
This would be helpful or useful	○	○
This would be possible/feasible	○	○

Section 2: General content

Do agree with the following suggestions for modifications?

11) Add more general resources based on region (e.g., support groups for depression or for caregivers, AA, mental health resources, CLSCs).

	Agree	Disagree
This would be helpful or useful	○	○
This would be possible/feasible	○	○

12) Add more questions to ask your healthcare team (specific diagnosis, what is my treatment plan, how will my family be involved, etc.) and how to advocate for your needs with your team.

	Agree	Disagree
This would be helpful or useful	○	○
This would be possible/feasible	○	○

13) Add more content on 'hope' to help address the feeling that nothing will change and include a reminder that it won't always feel this way and things can be done to help.

	Agree	Disagree
This would be helpful or useful	◯	◯
This would be possible/feasible	◯	◯

14) Add more information about anti-depressant medication (e.g., side-effects, mixing with alcohol, things to watch out for).

	Agree	Disagree
This would be helpful or useful	◯	◯
This would be possible/feasible	◯	◯

15) Verify the reading level of the material. If needed, edit so that it is accessible to those with a 5th grade reading level.

	Agree	Disagree
This would be helpful or useful	◯	◯
This would be possible/feasible	◯	◯

16) Include more tailoring of content to each person's needs. For example, try to include option so people can choose what is most relevant to them.

	Agree	Disagree
This would be helpful or useful	◯	◯
This would be possible/feasible	◯	◯

17) Change scales throughout the materials so they are all consistent (some 0-100, some 0-10).

	Agree	Disagree
This would be helpful or useful	◯	◯
This would be possible/feasible	◯	◯

18) In terms of 'reactivating your life,' it may be hard for some people to believe that activity can lead to feeling better. Add sentence with further justification (e.g., studies have shown…)

	Agree	Disagree
This would be helpful or useful	◯	◯
This would be possible/feasible	◯	◯

19) Add more content on de-stigmatizing depression.

	Agree	Disagree
This would be helpful or useful	◯	◯
This would be possible/feasible	◯	◯

20) Add more content for the person with the chronic condition about focusing on self-care and overcoming guilt about not being able to do certain things.

	Agree	Disagree
This would be helpful or useful	◯	◯
This would be possible/feasible	◯	◯

21) Reformulate some suggestions that are negatively framed (do not…) to be more positive (try this, do this, consider this).

	Agree	Disagree
This would be helpful or useful	◯	◯
This would be possible/feasible	◯	◯

22) Add a more detailed step-by-step introduction on how to use the materials in the toolkit.

	Agree	Disagree
This would be helpful or useful	◯	◯
This would be possible/feasible	◯	◯

23) Add more encouragement to get further help or support.

	Agree	Disagree
This would be helpful or useful	◯	◯
This would be possible/feasible	◯	◯

Section 3: Format

To what extent do agree with the following suggestions for modifications:

24) As few people were able to watch the DVD/video (it included info from HCPs on depression and testimonials), add written testimonials about depression.

	Agree	Disagree
This would be helpful or useful	○	○
This would be possible/feasible	○	○

25) Add more visual (graphic/images) representations of the written content.

	Agree	Disagree
This would be helpful or useful	○	○
This would be possible/feasible	○	○

26) Add more 'upbeat' colours.

	Agree	Disagree
This would be helpful or useful	○	○
This would be possible/feasible	○	○

27) Add further summaries of key points.

	Agree	Disagree
This would be helpful or useful	○	○
This would be possible/feasible	○	○

28) Increase type face (font) size and verify that type face (font) is the most accessible to readers.

	Agree	Disagree
This would be helpful or useful	○	○
This would be possible/feasible	○	○

29) Add visual component (like a Powerpoint) to go with the audio version (CD) of the workbook.

	Agree	Disagree
This would be helpful or useful	○	○
This would be possible/feasible	○	○

30) Use audio format in which the speed can be adjusted (faster or slower pace).

	Agree	Disagree
This would be helpful or useful	○	○

	Agree	Disagree
This would be possible/feasible	○	○

31) Develop online version that is more interactive.

	Agree	Disagree
This would be helpful or useful	○	○
This would be possible/feasible	○	○

32) Consider online blog or chat group component so people can connect/interact with others with shared experience.

	Agree	Disagree
This would be helpful or useful	○	○
This would be possible/feasible	○	○

33) Include an additional workbook with more activities based on the skills presented.

	Agree	Disagree
This would be helpful or useful	○	○
This would be possible/feasible	○	○

34) Add an online mood monitoring (mood tracker) that can graphically display trends.

	Agree	Disagree
This would be helpful or useful	○	○
This would be possible/feasible	○	○

35) Have online mood tracker followed by healthcare team who can follow-up.

	Agree	Disagree
This would be helpful or useful	○	○
This would be possible/feasible	○	○

Do you have any other comments or feedback?
